# Neuroendocrine Carcinoma of the Stomach: A Case Study

**DOI:** 10.1155/2011/948328

**Published:** 2011-12-14

**Authors:** Keisuke Kubota, Akihiro Okada, Junko Kuroda, Masashi Yoshida, Keiichiro Ohta, Miki Adachi, Masayuki Itabashi, Yoshiyuki Osamura, Masaki Kitajima

**Affiliations:** ^1^Department of Gastroenterological Surgery, International University of Health and Welfare Mita Hospital, 1-4-3 Mita, Minato-ku, Tokyo 108-8329, Japan; ^2^Department of Pathology, International University of Health and Welfare Mita Hospital, 1-4-3 Mita, Minato-ku, Tokyo 108-8329, Japan

## Abstract

Gastric neuroendocrine carcinomas are rare and have a poor prognosis, and the diagnostic criteria for this disease have recently changed. We herein report a case of sporadic gastric neuroendocrine carcinoma. A 75-year-old man was referred to our hospital with epigastric pain. Endoscopic examination revealed a localized ulcerative lesion (diameter, 4 cm) at the upper stomach. The diagnosis on biopsy was neuroendocrine carcinoma. Total gastrectomy with D2 lymphadenectomy, splenectomy, and cholecystectomy was performed. Pathologically, the tumor infiltrated the subserosal layer, and 6/49 lymph nodes were involved. The tumor was uniform in shape and arranged in a rosette-like structure to form solid nests, with medium-sized, round-to-cuboid-shaped tumor cells and intense mitosis 46/10 HPF. It was positive for synaptophysin and chromogranin A, and the Ki-67 labeling index was 70–80%. The diagnosis of neuroendocrine carcinoma was made according to the WHO 2010 criteria. The patient was followed up for three years without recurrence.

## 1. Introduction

Neuroendocrine carcinomas (NECs) of the stomach, although rare, deserve particular attention as they are aggressive and have an extremely poor prognosis [1–6]. In addition, the concept of this disease and its diagnostic criteria have been changed recently. The World Health Organization (WHO) proposed new diagnostic criteria in 2010 that mainly depend on the rate of cellular proliferation [7].

 In this paper, we describe a case of sporadic gastric NEC with a fine outcome. We also describe novel suggestions for the diagnosis of gastric NEC.

## 2. Case Presentation

**Figure 1:**
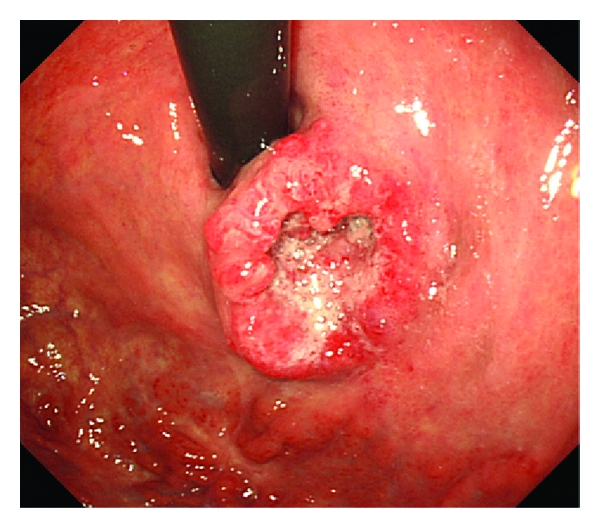
Upper endoscopic examination revealed a localized ulcerative lesion (diameter, 4 cm) located on the lesser curvature of the upper stomach.

**Figure 2:**
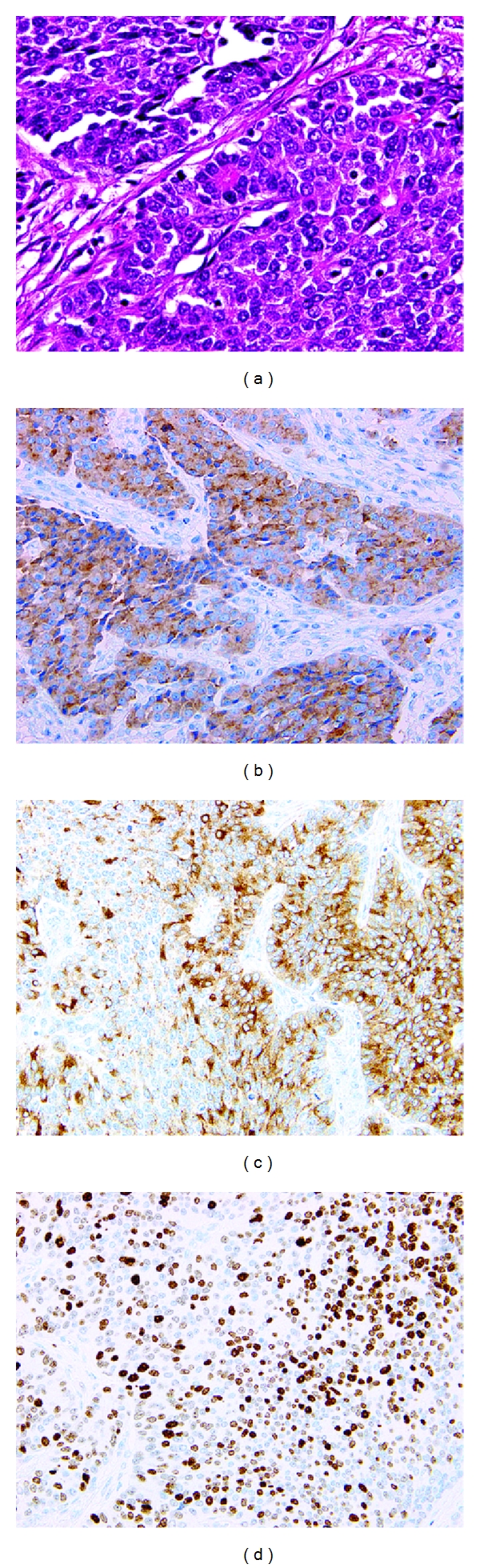
Histological findings of the tumor (x 400). The tumor was uniform in shape and arranged in small microtubular structures (rosette-like arrangement) to form solid nests, with medium-sized, round-to-cuboid-shaped tumor cells. The tumor cells exhibited intense mitosis greater than 2/HPF (hematoxylin and eosin, (a)). Immunohistochemical staining showed that it was positive for synaptophysin (b) and chromogranin A (c). The Ki-67 labeling index was 70–80% (d).

A 75-year-old man was referred to our hospital with left epigastric pain. Upper endoscopic examination revealed a localized ulcerative lesion (diameter, 4 cm) located on the lesser curvature of the upper stomach (Figure 1). The tumor was thought to invade the subserosal layer. A biopsy of the lesion gave the diagnosis of NEC. Patient interview revealed no particular past history or familial history. No extragastric hormonal syndromes, such as flushes or diarrhea, were identified. After obtaining informed consent, a total gastrectomy with D2 lymphadenectomy, splenectomy, and cholecystectomy was performed [8]. Pathologically, the tumor was 30 × 27 mm in size and with a negative margin. The tumor infiltrated the subserosal layer. Microscopically, the tumor was uniform in shape and arranged in small microtubular structures (rosette-like arrangement) to form solid nests, with medium-sized, round-to-cuboid-shaped tumor cells containing clear and rich cytoplasm. The tumor cells exhibited hyperchromatic nuclei and intense mitosis 46/10 HPF (Figure 2(a)). Lymphatic invasion was widely observed and lymph node involvement was seen in 6/49 nodes. By immunohistochemical staining, the tumor cells were positive for synaptophysin (Figure 2(b)), chromogranin A (Figure 2(c)), and CEA and negative for S-100 protein. The Ki-67 labeling index was 70–80% (Figure 2(d)). These findings led to the diagnosis of NEC of the large cell type according to the 2010 WHO criteria [7]. The postoperative course was uneventful, and the patient was followed up without any maintenance therapy for three years without any findings indicative of recurrence or distant metastasis.

## 3. Discussion

Gastric neuroendocrine neoplasms (NENs) embrace a group of tumors that exhibit a spectrum of histopathologic variations, ranging from clearly benign tumors to highly malignant ones. Recently, the concept of this disease and its diagnostic criteria have been changed. In the 2010 WHO criteria, NENs of the stomach are defined as neoplasms with neuroendocrine differentiation, including neuroendocrine tumors (NETs) and NECs arising in the stomach [7]. Synonyms for gastric NETs include carcinoid, well-differentiated endocrine tumor/carcinomas, and enterochromaffin-like cell NETs, and synonyms for NECs include poorly differentiated endocrine carcinomas and small cell and large cell endocrine carcinomas. NENs are classified into NET G1 (carcinoid) and G2, NECs, mixed adenoneuroendocrine carcinomas, enterochromaffin cells, serotonin-producing NETs, and gastrin-producing NETs [7]. In the Japanese Classification of Gastric Carcinoma, NENs are classified into carcinoid tumors and endocrine carcinomas (small cell type and large cell type) [8].

Although the prevalence of gastric NENs has recently risen, they are thought to be relatively rare tumors that account for less than 1% of all gastric tumors [9]. In general, the majority of these tumors are NETs, whose courses are indolent and not life threatening. Concerning NETs, more than 100 years have passed since Oberndorfer proposed the term “carcinoid” in 1907 [10]. In 1993, Rindi et al. advocated a classification system with three subtypes of gastric carcinoid tumors according to the clinicopathological features [1], and this classification system is reflected in the 2010 WHO criteria. On this background, Gilligan et al. advocated a treatment algorithm for gastric carcinoid tumors, including the above-mentioned subtypes as well as the size and number of tumors [11]. Recently, less invasive therapeutic options, such as endoscopic resection of the tumor, have been reported for small NETs [12].

NECs of the stomach are also rare, representing less than 10% of gastric NENs [2, 13], and such rarity has made it difficult to understand precisely their biological nature and to establish optimal treatment options. The NEC of our case was a difficult diagnosis to establish, and the immunohistochemistry played a major role. Microscopically, the patient's tumor was uniform in shape and arranged in small microtubular structures (rosette-like arrangement) to form solid nests, with medium-sized, round-to-cuboid-shaped tumor cells on hematoxylin and eosin staining. On immunohistochemistry, the tumors are usually positive for synaptophysin and neuronal-specific enolase, but are rarely positive for the chromogranin A staining observed in our patient. In the 2010 WHO criteria, NENs are classified into NETs or NECs on the basis of the level of cellular proliferation, including the mitotic and Ki-67 indices. The mitotic index was 46/10 HPF and the Ki-67 labeling index was 70–80% in our case, so he was diagnosed as NEC. Lymphatic invasion was widely observed and lymph node involvement was seen in many nodes, suggesting the high-grade malignant nature of this tumor and showing the compatibility of the diagnosis. Aggressive surgery and chemotherapy should be considered for any NEC [3]. A total gastrectomy with D2 lymphadenectomy, splenectomy, and cholecystectomy was performed on our patient, and he has lived disease-free for three years without any maintenance therapy.

In conclusion, we described a case of sporadic gastric NEC. An adequate description of NECs should be globally and historically discussed in relation to the real manifestation of this tumor group, considering the evaluation of the Consensus Conference. The diagnosis and treatment of these tumors should be evaluated in large clinical studies.

## Abbreviations

NEC: Neuroendocrine carcinoma
NEN: Neuroendocrine neoplasm
NET: Neuroendocrine tumor
WHO: World Health Organization.

## References

[1] Rindi G, Luinetti O, Cornaggia M, Capella C, Solcia E (1993). Three subtypes of gastric argyrophil carcinoid and the gastric neuroendocrine carcinoma: a clinicopathologic study. *Gastroenterology*.

[2] Rindi G, Bordi C, Rappel S, la Rosa S, Stolte M, Solcia E (1996). Gastric carcinoids and neuroendocrine carcinomas: pathogenesis, pathology, and behavior. *World Journal of Surgery*.

[3] Rindi G (1995). Clinicopathologic aspects of gastric neuroendocrine tumors. *American Journal of Surgical Pathology*.

[4] Waisberg J, de Matos LL, Mader AMDAA (2006). Neuroendocrine gastric carcinoma expressing somatostatin: a highly malignant, rare tumor. *World Journal of Gastroenterology*.

[5] Matsui K, Jin XM, Kitagawa M, Miwa A (1998). Clinicopathologic features of neuroendocrine carcinomas of the stomach: appraisal of small cell and large cell variants. *Archives of Pathology and Laboratory Medicine*.

[6] Yu JY, Wang LP, Meng YH, Hu M, Wang JL, Bordi C (1998). Classification of gastric neuroendocrine tumors and its clinicopathologic significance. *World Journal of Gastroenterology*.

[7] Bosman FT, Carneiro F, Hruban RH, Theise ND (2010). *WHO Classification of Tumors of the Digestive System*.

[8] Japanese Gastric Cancer Association (2010). *Japanese Classification of Gastric Carcinoma (in Japanese)*.

[9] Modlin IM, Lye KD, Kidd M (2003). Carcinoid tumors of the stomach. *Surgical Oncology*.

[10] Oberndorfer S (1907). Karzinoide tumoren des duendarms. *Frankfurter Zeitschrift für Pathologie*.

[11] Gilligan CJ, Lawton GP, Tang LH, West AB, Modlin IM (1995). Gastric carcinoid tumors: the biology and therapy of an enigmatic and controversial lesion. *American Journal of Gastroenterology*.

[12] Simoyama S, Fujishiro M, Takazawa Y (2010). Successful type-oriented endoscopic resection for gastric carcinoid tumors: a case report. *World Journal of Gastrointestinal Endoscopy*.

[13] Chiba N, Suwa T, Hori M, Sakuma M, Kitajima M (2004). Advanced gastric endocrine cell carcinona with distant lymph node metastasis: a case report and clinicopathological characteristics of the disease. *Gastric Cancer*.

